# Haemophilus influenzae Type f Meningitis Complicated by Bilateral Subdural Empyema, Central Venous Thrombosis, and Bilateral Sensorineural Hearing Loss in an Immunocompetent 12-month-old

**DOI:** 10.7759/cureus.4850

**Published:** 2019-06-06

**Authors:** Jacob Abou-Hanna, Katherine Panning, Hiral Mehta

**Affiliations:** 1 Pediatrics, University of Michigan Medical School, Ann Arbor, USA; 2 Pediatrics, C.S. Mott Children's Hospital, Ann Arbor, USA

**Keywords:** meningitis, haemophilus influenzae, subdural empyema, hib vaccine, sensorineural hearing loss

## Abstract

Haemophilus influenzae is a gram-negative coccobacillus that colonizes the nasopharyngeal surface and upper respiratory tract of healthy individuals and includes six encapsulated serotypes as well as non-encapsulated, non-typeable strains. Since the widespread use of the Haemophilus influenzae type b (Hib) conjugate vaccine implemented in 1990, the majority of invasive illness now seen in the United States is secondary to capsular serotypes other than type b and non-typeable strains with the largest burden of disease affecting the extremes of age-infants and the elderly. We report a case of acute Haemophilus influenzae type f meningitis in a 12-month-old female who was previously healthy and had been fully immunized. She demonstrated clinical improvement on ceftriaxone, but persistent fever and ear-tugging resulted in obtaining an MRI that displayed bilateral subdural empyemas requiring burr-hole craniotomy, central venous thrombosis requiring anticoagulation, and bilateral sensorineural hearing loss requiring cochlear implants. Immunological studies confirmed immunocompetency and appropriate response to her previous Hib vaccination, suggesting a significant impact of bacterial virulence. These complications, with the exception of sensorineural hearing loss, have not been reported in the literature for Haemophilus influenzae type f and should be considered in the care of these patients despite clinical appearance given the severity of complications and potential for acute decompensation. Despite the success of vaccination in reducing invasive disease, cases of H. influenzae meningitis continue to occur via less common encapsulated serotypes with unknown complications, making the management and treatment of these infections more difficult for practitioners.

## Introduction

Haemophilus influenzae is a gram-negative coccobacillus that colonizes the nasopharyngeal surface and upper respiratory tract of healthy individuals and includes six encapsulated serotypes as well as non-encapsulated, non-typeable strains [1, 2]. Since the widespread use of the Haemophilus influenzae type b (Hib) conjugate vaccine implemented in 1990, the majority of invasive illness in the United States is secondary to capsular serotypes other than type b and non-typeable strains with the largest burden of disease affecting the extremes of age-infants and the elderly [3-5]. We report a case of acute bacterial meningitis in a previously healthy, fully immunized 12-month-old female. The pathogen was identified as Haemophilus influenzae type f and was successfully treated with a four-week course of ceftriaxone.

## Case presentation

A 12-month-old, previously healthy, fully immunized female developed cough, congestion, vomiting, and fever with poor oral intake and decreased urine output leading to first presentation at the emergency department. She showed improvement in activity level during the evaluation and was discharged home with a clinical diagnosis of a viral upper respiratory infection. Over the next four days, she had normal oral intake and urine output, but continued to have decreased activity level, congestion, mild cough, and fever despite scheduled antipyretics. Her parents noted that she was fussy, refused to sit up, and was not acting like herself. She developed increased work of breathing which prompted a second presentation to the emergency department. She was noted to be afebrile, awake, and alert but was significantly irritable with nuchal rigidity. Cranial imaging was not performed, but lumbar puncture revealed cloudy cerebrospinal fluid (CSF) concerning for bacterial meningitis. Initial CSF studies revealed: Glucose <4 mg/dL, protein 107 mg/dL, Leukocyte count 4,063 cells/cmm, and 89% neutrophils. She was empirically started on ceftriaxone and vancomycin and admitted for bacterial meningitis.

**Figure 1:**
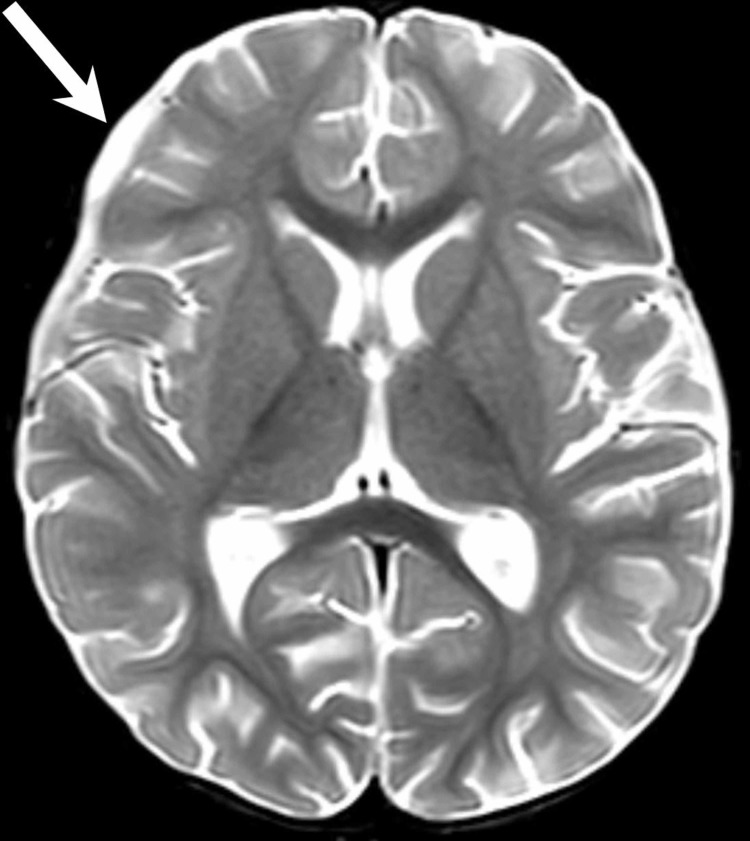
Axial, T2-weighted MRI showing bilateral subdural empyemas over the bilateral cerebral convexities most prominent in the right frontal region (arrow) which required right burr-hole craniotomy.

CSF cultures eventually grew H. influenzae type f sensitive to ceftriaxone. Respiratory swab revealed adenovirus co-infection. She improved clinically in terms of alertness and activity level with down-trending leukocytosis and inflammatory markers but continued to spike fevers despite scheduled antipyretics and was noted to be tugging at her right ear. A magnetic resonance imaging (MRI) scan was obtained, which showed bilateral subdural empyema (SDE), central venous thrombosis, and enhancement of inner ears (Figure 1).

**Figure 2:**
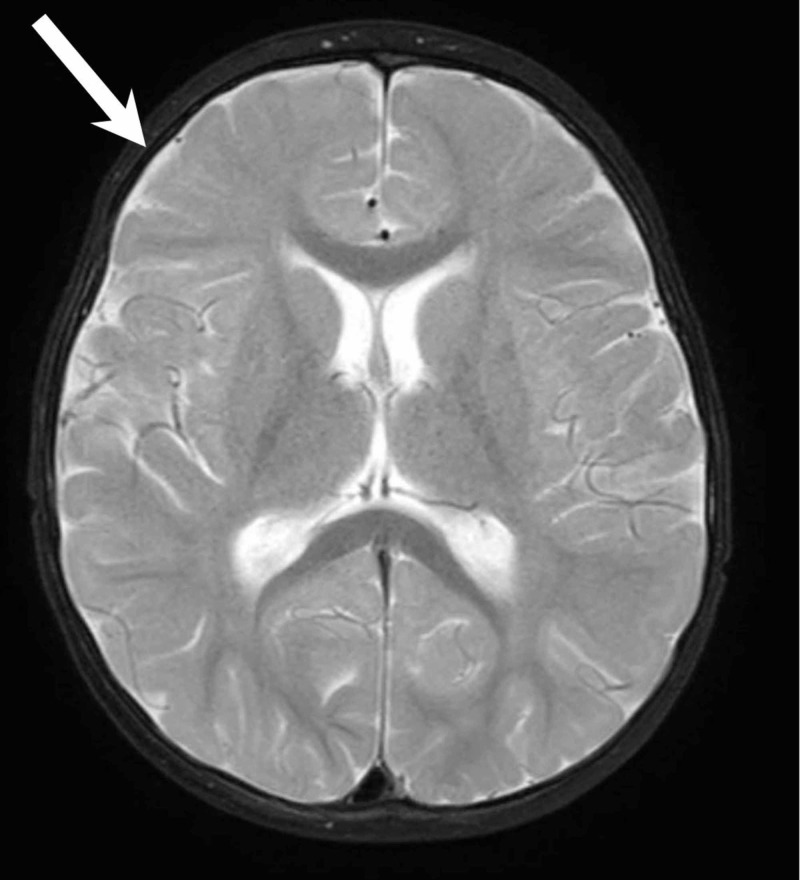
Axial, T2-weighted MRI showing resolution of bilateral subdural empyemas (arrow) as compared to the previous figure.

**Figure 3:**
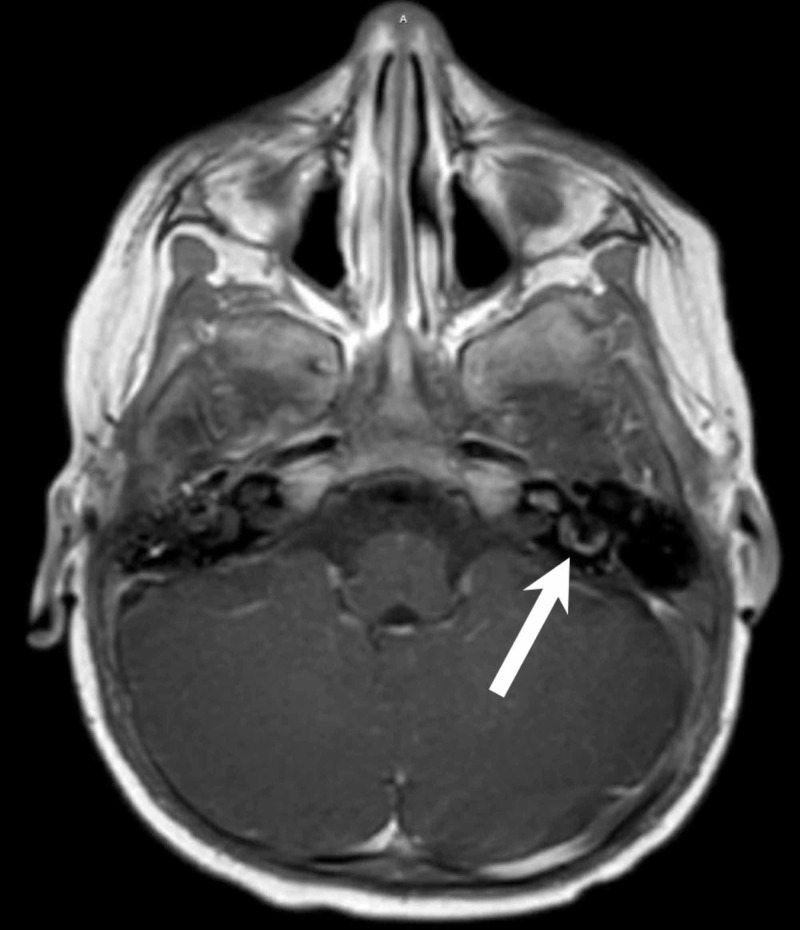
Axial, post-contrast T1-weighted MRI displaying persistent labyrinthitis (arrow).

Right-sided burr-hole craniotomy was performed to drain the empyema for further source control; cultures of the purulent drainage were negative. The central venous thrombosis was treated with heparin infusion with transition to enoxaparin. Repeat MRI showed improving empyema (Figure 2), but persistent labyrinthitis (Figure 3). Given the severity of her presentation, immunological studies (Table 1) were performed and confirmed she was immunocompetent and had responded to her previous Hib vaccination. To assess for functional asplenia, a blood smear was performed and showed no Howell-Jolly bodies.

**Table 1:** Immunologic studies demonstrating appropriate response to Hib vaccine and normal range total antibodies and complement levels.

Component	Value	Reference Value
H. Influenzae B IgG	3.37	>= 0.15 mg/L
Total IgG	569	280-1,030 mg/dL
Total IgA	33	15-160 mg/dL
Total IgM	108	35-140 mg/dL
Total Complement, CH50	73	41-95 U/mL
Total Complement, AH50	66	>=46%
Tetanus IgG	0.30	>=0.01 IU/mL
Diphtheria IgG	0.08	>=0.01 IU/mL

Intravenous antibiotics were administered for four weeks and discontinued based on resolution of findings previously seen on MRI. Enoxaparin was administered for a total of six weeks. Auditory Brainstem Response testing performed during repeat MRI showed profound bilateral sensorineural hearing loss, requiring bilateral cochlear implants and tympanostomy tubes.

## Discussion

We report a case of a 12-month-old, immunocompetent female that presented with Haemophilus influenza type f meningitis and a clinical course complicated by bilateral subdural empyemas, central venous thrombosis, and bilateral sensorineural hearing loss. Her initial presentation was significant for viral infection positive for adenovirus and recent history of bilateral acute otitis media which may have predisposed her to colonization and infection [6, 7]. Apart from sensorineural hearing loss, these complications have not been reported in the literature for Haemophilus influenzae type f [8, 9] and should be considered in the care of these patients despite clinical appearance given the severity of complications and potential for acute decompensation. In this case, our patient showed clinical improvement in terms of temperament, behavior, and nuchal rigidity after IV antibiotic administration as well as down-trending inflammatory markers. However, subtle findings of ear tugging and persistent fever prompted further investigation leading to MRI imaging and further source control. This serves as a reminder for providers to maintain a high index of suspicion for complications in cases of bacterial meningitis, such as SDE, as symptoms may be subtle [10].

In infants, bacterial meningitis is the main cause of SDE [10]. Up to 2% of patients with bacterial meningitis will develop SDE [11]. The main features are fever, seizure, and impaired consciousness, which is difficult to discern from meningitis. Thus, imaging is needed to diagnose SDE; cranial ultrasonography is feasible in low resource settings, while high resolution contrast CT and contrast enhanced MRI are the gold standard for diagnosis [12]. Further considerations in the treatment of SDE include a prolonged antibiotic course as well as surgical intervention. Surgical strategies include burr hole drainage as a less invasive approach with comparable clinical outcomes to craniotomy [11].

Non-type b serotypes of Haemophilus influenza, notably serotype f, are growing in prevalence [13, 14]. In children, pneumonia and meningitis are the most common forms of type f infection, while pneumonia is most common in adults [15]. Additionally, the majority of invasive non-type b Hi cases are seen in immunocompetent patients with no predisposing factors, thereby suggesting a significant impact of bacterial virulence [16]. In response to these growing numbers, non-typable, broad coverage Hi vaccines are currently being developed [17, 18]. In the meantime, this report aims to contribute to the understanding of this decreasingly rare disease, thereby helping in management of these patients.

## Conclusions

Despite the success of vaccination in reducing invasive disease, cases of H. influenzae meningitis continue to occur via less common encapsulated serotypes with unknown complications making the management and treatment of these infections more difficult for practitioners. Continued reporting of these non-type b cases will aid in achieving further understanding and appropriate management.
